# Supplementary material for: Content-rich biological network constructed by mining PubMed abstracts
Source: BMC Bioinformatics. 2004 Oct 8;5:147. doi: 10.1186/1471-2105-5-147 (PMC528731; doi:10.1186/1471-2105-5-147)
Supplement: Additional File 2 — The original results of the above study (non-essential files are deleted to keep the file size under the limit set by BMC bioinformatics). [file 1471-2105-5-147-S2.bz2 › chilibotAdditionalFile2/dip05/417ID7673243E10286/html/BMP4_BMPR1B.html]

 


 **BMP4** and **BMPR1B** 
  
Found 11 abstracts in PubMed, retrieved 05.  
 

 What does Google say? 
 PDF only 
| .edu only 

---

**Interactive relationship** (e.g. stimulation, inhibition, etc)

**Neutral relationship**- BMP 4  [ **BMP4** ]  and GDF 5 are known to bind to activin receptor like kinase 3 ALK 3 and or ALK 6  [ **BMPR1B** ]  also termed BMP type IA and type IB receptors, respectively.  Ref: 11282024 J Cell Sci, 2001
- In the early stage, BMP 4  [ **BMP4** ]  derived mRNA was localized mainly in cells in the anterior margin of the cervical discs, together with ALK 6  [ **BMPR1B** ]  and BMP RII mRNA.  Ref: 11147870 J Neurosurg, 2001
- In transfection experiments with HEK 293 cells, co expression of FecB  **BMPR1B**  and BMPR2 resulted in a 2.6 fold P< ; 0.01 induction of the activity of a BMP specific luciferase reporter construct by BMP 4  [ **BMP4** ] .  Ref: 12773124 J Endocrinol, 2003

**Non-interactive relationship** (e.g. studied together, co-existance, homology, etc.)

- To evaluate the consequences of the Booroola mutation on  **BMPR1B**  functions, the action of ligands of the transforming growth factor beta TGFbeta BMP family that act through growth and differentiation factor 5, BMP 4  [ **BMP4** ]  or independently of activin A, TGFbeta 1  **BMPR1B**  were studied on primary cultures of GCs from homozygous FecB and FecB B ewes.  Ref: 12773124 J Endocrinol, 2003
- Interestingly, no response to BMP 4  [ **BMP4** ]  was observed when cells were transfected with the FecB B form of the  **BMPR1B**  receptor.  Ref: 12773124 J Endocrinol, 2003
